# Research on wheel-legged robot based on LQR and ADRC

**DOI:** 10.1038/s41598-023-41462-1

**Published:** 2023-09-13

**Authors:** Xujiong Feng, Shuaishuai Liu, Qiang Yuan, Junbo Xiao, Daxu Zhao

**Affiliations:** 1 Jiangsu Key Laboratory of Advanced Manufacturing Technology, Huaiyin Institute of Technology, Jiangsu, 223003 China; 2 Zhejiang AF University, Hangzhou, 310000 China

**Keywords:** Electrical and electronic engineering, Mechanical engineering

## Abstract

The traditional two-wheeled self-balancing robot can travel quickly in a flat road environment, and it is easy to destabilize and capsize when passing through a bumpy road. To improve the passing ability of a two-wheeled robot, a new wheel-legged two-wheeled robot is developed. A seven-link leg structure is proposed through the comprehensive design of mechanism configuration, which decouples the balanced motion and leg motion of the robot. Based on the Euler–Lagrange method, the dynamic model of the system is obtained by applying the nonholonomic dynamic Routh equation in the generalized coordinate system. The robot’s state space is divided according to the robot’s height, and the Riccati equation is solved in real-time by the linear quadratic regulator (LQR) method to complete the balance and motion control of the robot. The robot leg motion control is achieved based on the active disturbance rejection control (ADRC) way. A robot simulation model is built on Recurdyn to verify the algorithm’s feasibility, and then an experimental prototype is built to demonstrate the algorithm’s effectiveness. The experimental results show that the control method based on LQR and ADRC can make the robot pass through the bumpy road.

## Introduction

**Figure 1:**
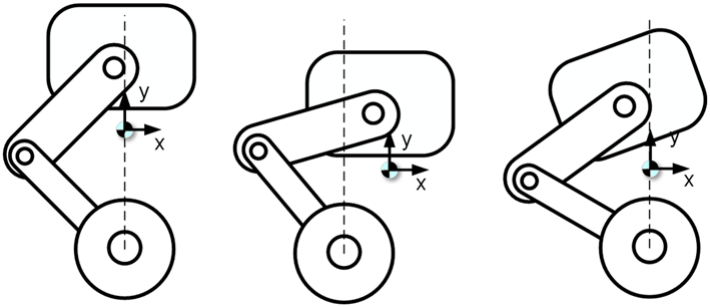
Centroid offset diagram.

The traditional two-wheeled self-balancing robot has the advantages of small size^1, 2^, lightweight and flexible movement, but the environmental conditions on the ground limit it. To move the two-wheeled robot not limited to the flat road environment, the wheel-legged two-wheeled self-balancing robot came into being. Compared with traditional self-balancing robots, wheel-legged self-balancing robots increase the degree of freedom of the legs. When the leg mechanism moves, the robot’s center of mass will shift^3^, decreasing the effect of the traditional control strategy^4, 5^. In Fig. 1, for the wheel-legged two-wheeled robot, to reduce the influence of leg motion on the balance control of the robot, when the leg’s centroid trajectory should be ensured to pass through the two-wheel axis vertically as much as possible. The attitude angle of the platform on the vehicle should stay the same as possible. Because in many applications, the body is expected to remainstable, such as handling agricultural products.

To improve the robustness of the wheel-legged robot, Wang^6^ proposed a series of double closed-loop control strategies combining ADRC^7, 8^ and PID^9–11^ control, enhancing the robot’s robustness in fixed-point balance. However, there are still challenges in the speed control of the robot. Shahida Khatoon et al.^12^ designed LQR^13^ and model predictive control (MPC)^14–16^ controllers for two-wheeled self-balancing robots. However, due to the introduction of the degree of freedom of the legs, it is not straightforward to model and linearize the robot dynamics. Ollie^17^ decoupled the robot’s balanced leg motion through a parallel five-bar leg geometry and added a robotic arm assist to assist the robot in completing jumps such as backflips. However, the robot is controlled by seven motors, with complex driving and low energy utilization.

**Figure 2:**
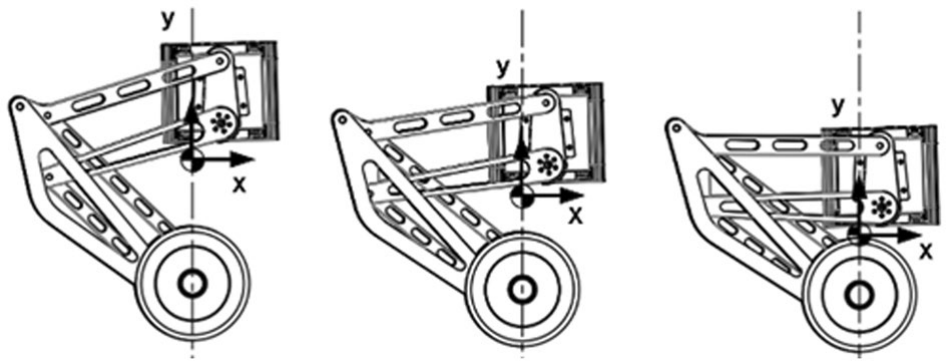
Wheel-legged robot squat motion centroid change diagram.

In order to solve the problem of centroid deviation during robot movement, a seven-link leg geometry is proposed. As shown in Fig. 2, it limits the offset of the robot’s centroid in the X-axis direction and decouples the robot’s balanced and leg movements. For the balance of the center of mass in the Y-axis direction, it is divided into different state spaces according to the different heights of the robot’s center of mass. Then the optimal feedback control rate is obtained by solving the Riccati equation in realtime, which depends on the robot leg feedback for a stable and accurate center of mass height. Therefore, the active disturbance rejection method is used to complete the control of the robot leg. ADRC makes the robot legs have a robust anti-interference ability to provide a stable centroid height for the LQR controller. LQR and ADRC re-simulation through the test and in the experimental prototype to complete the experiment, reflecting the robustness of the control strategy.

The main contributions of this paper are as follows:

1. Robot dynamics modeling and robot controller design;
2. Robot mechanical structure design and hardware implementation;
3. The simulation model and the actual test prototype are established to verify the feasibility and effectiveness of the control method.

The following structure will be used for analysis: The second chapter introduces the robot system’s structure design and hardware implementation. In the third chapter, based on the Euler–Lagrange method, the system dynamics model is obtained by applying the nonholonomic Routh equation in the generalized coordinate system. The fourth chapter focuses on the design of the robot controller. Chapter 5 shows the performance of robots in simulated and real-world.

## System description

**Figure 3:**
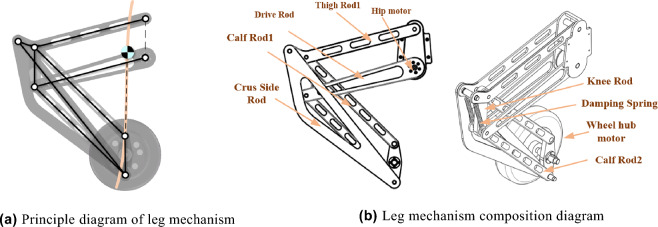
Leg mechanism diagram.

As shown in Fig. 3a, the wheel leg structure of the robot is a seven-link mechanism. The mechanism has one degree of freedom, allowing only upward movement of the legs. This parallelogram structure can ensure that the robot’s body Angle does not change during the ascent process. To maximize the decoupling of leg movement and robot balance movement. Taking the distance of the centroid offset in the X-axis as the optimization objective, the least square method was used to determine the optimal length of each connecting rod through the constraints of the plane’s mechanism. The trajectory of the robot’s center of mass is an approximately straight line perpendicular to the hub motor’s axis in the travel range, which reduces the deviation error of the center of gravity during the robot’s leg movement.

As shown in Fig. 3b, the drive rod and the body frame are connected by a hip motor with a harmonic reducer. The FOC algorithm drives the hip motor, and the harmonic reducer makes the motor peak torque up to 80 NM. A damping spring is added between the thigh rod and the calf rod. On the one hand, the tension of the energy storage spring offsets the weight of the system itself, reducing the pressure of the system gravity on the hip joint motor, and on the other hand, it plays a shock absorber role during driving. At the end of the leg is a 15 NM wheel hub motor (Sentron 10530), which is responsible for the robot’s fixed-point balance and driving.

**Figure 4:**
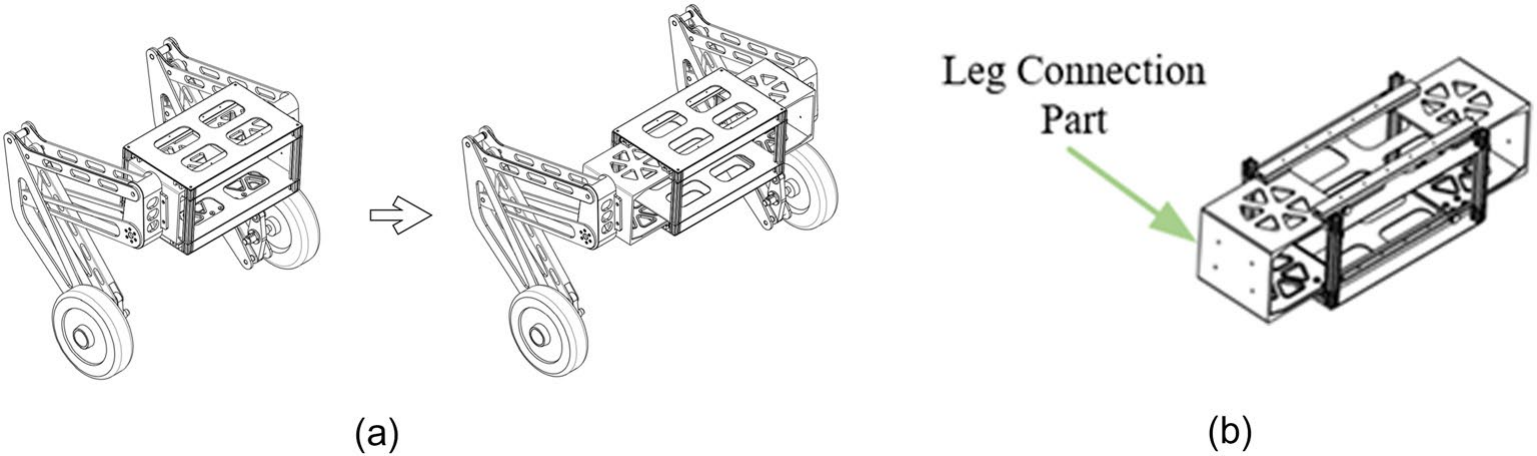
Body structure diagram.

As shown in Fig. 4, the robot’s body is a transversely retractable rectangular structure. The span of the wheel legs can be adjusted to adapt to different environments through the expansion of the connecting parts. The leading equipment and sensors are installed in the body part, including Single-chip Microcomputer (SCM) (STM32H743IIT6), power, Inertial Measurement (IMU)(HWT605), etc.

**Figure 5:**
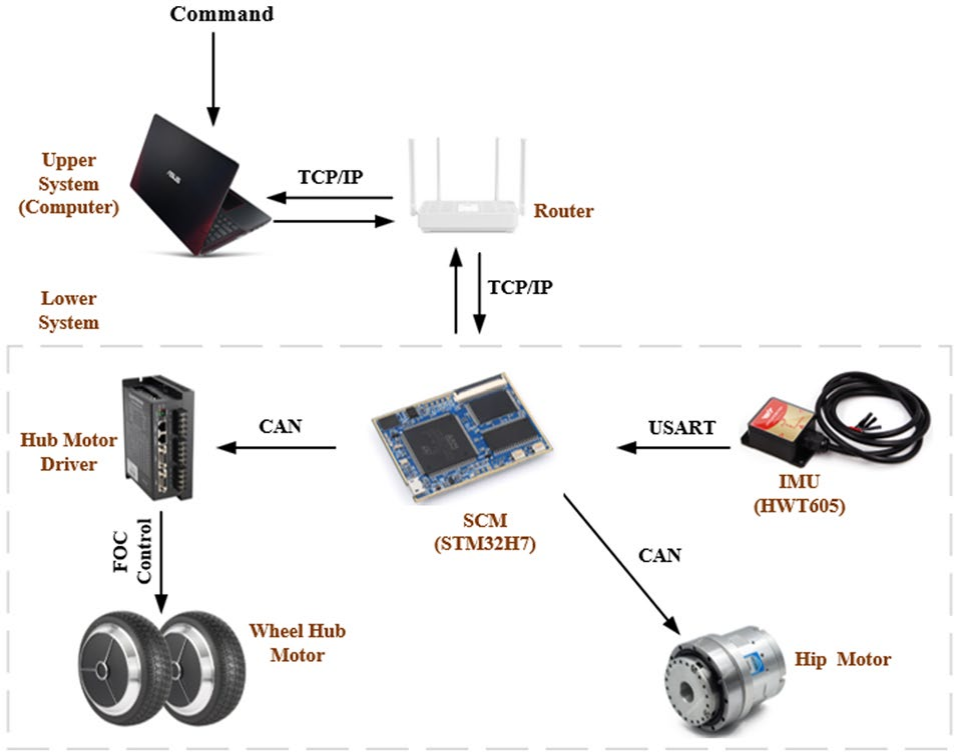
System communication diagram.

In Fig. 5, the hub motor and hip motor communicate with SCM through the CAN bus to provide feedback on the robot’s speed, position and height information. The IMU sends the robot’s attitude information to the processor through the serial port. The processor receives the information to complete the robot control and other work. Meanwhile, the main control board connects with the computer through the local area network to realize the wireless communication between the upper computer and the wheel-legged machine.

### Dynamic model

Compared with the traditional two-wheeled self-balancing robot, the wheel-legged robot increases the leg’s degree of freedom of movement to actively adjust the body’s height. On the other hand, it also makes the system more complex. Because the optimal design of the leg geometry decouples the robot’s driving and leg motion, the robot dynamics model is divided into the body and leg dynamics models.

#### Body dynamics model

**Figure 6:**
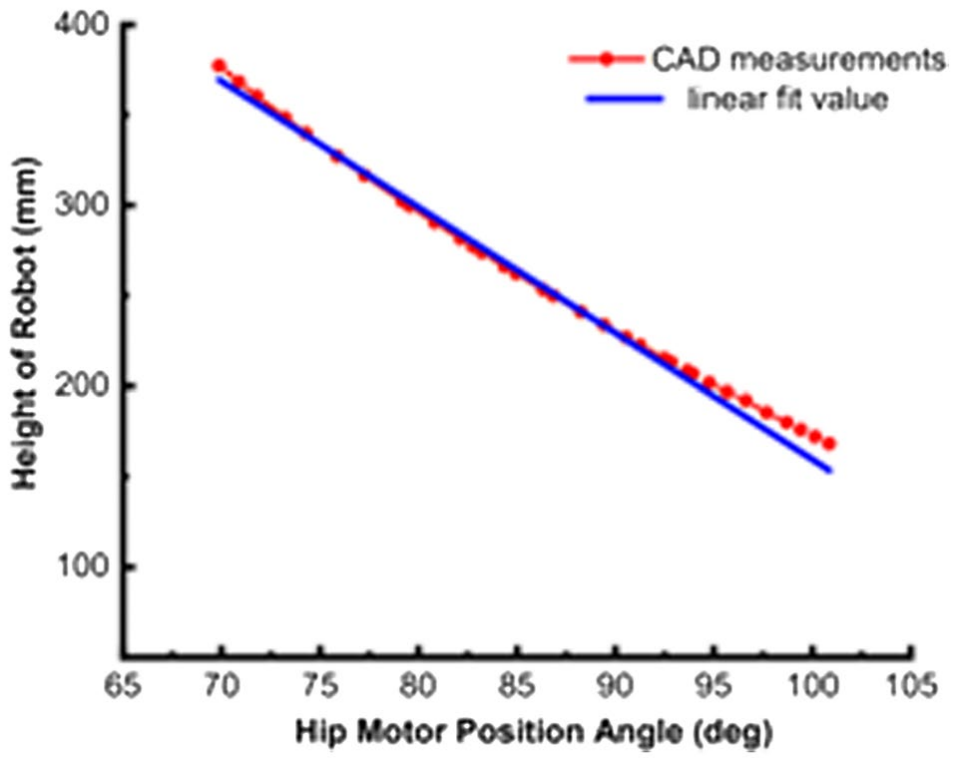
Relation diagram of robot height and hip joint motor angle.

For the body part, when the hip joint motor is locked, the body and the leg structure are regarded as one, which is equivalent to a single-stage inverted pendulum with a fixed center of mass. When the hip joint moves, it is analogous to a single-stage inverted pendulum whose centroid position changes in the vertical direction. As shown in Fig. 6, the robot’s overall centroid height is linear with the hip joint angle. The relationship between the robot’s centroid and the hip joint motor angle is measured by computer-aided measurement and then obtained by linear fitting.

The relationship between the height of the robot’s center of mass and the hip joint angle is obtained by fitting after measurement:

1 $$L_{m} = {831}.{37} - {6}.{64}\phi$$

**Figure 7:**
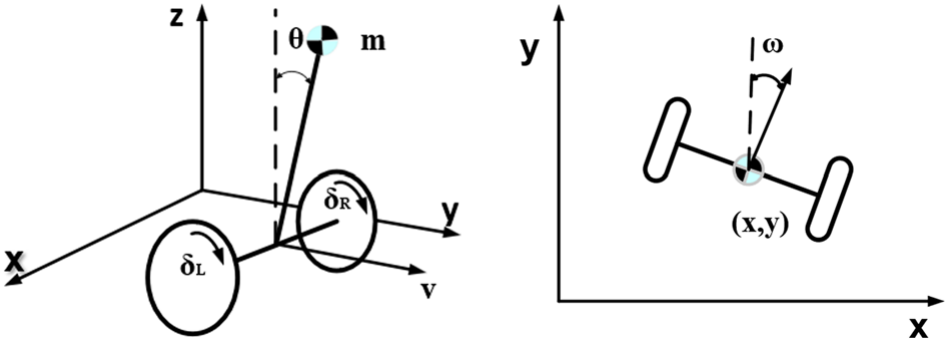
System schematic.

As shown in Fig. 7, when the hip joint angle is constant, the wheel-legged robot body dynamics model can be simplified to a highly fixed two-wheel inverted pendulum model. The generalized coordinate of the system $q = [ x\; \;y\;\;\theta \;\;\omega \;\;\delta_{L} \;\;\delta_{R} ]^{T}$ is selected, and the dynamic model of the wheel-legged two-wheel robot is constructed by the Euler–Lagrange method.

In Fig. 7 θ is the body pitch angle. ω is the body yaw angle. *v* is the robot’s linear velocity. *x* and *y* are x-axis and y-axis displacements. δ_*L*_ and δ_*R*_ are the rotation angles of the left and right wheels of the robot. In order to reduce the workload of modeling and simplify the model, the following assumptions need to be made:

1. pure rolling without sliding between wheel and ground;
2. The dynamics of the leg linkage mechanism are not considered;
3. All objects are rigid and have a uniform density.

Each component’s kinetic and potential energy are obtained using the space coordinate velocity transfer formula, and the Lagrangian energy function is obtained.

2 $$L = T - V$$

According to the Euler–Lagrange method, the nonholonomic dynamic Routh equation applied in the generalized coordinate system is:

3 $$\frac{d}{dt}\left( {\frac{\partial L}{{\partial q}}} \right) - \frac{\partial L}{{\partial q}} = Q + F_{T}(q)\lambda$$

From Formula 3:

4 $$M(q) \ddot{q} + N(q, \dot{q}) = J(q)\tau + F_{T}(q)\lambda$$

where *M*(*q*) is the mass matrix, $N(q,\dot{q})$ is the Coriolis gravity term; *J*(*q*) is Jacobian matrix; τ is the input vector; *F*_*T*_ (*q*) is a nonholonomic constraint matrix; λ for the Lagrangian multiplier.

The *F*_*T*_ (*q*) matrix is obtained by the two-wheel-legged robot subject to nonholonomic constraints. The Lagrange multiplier λ is eliminated according to the null space of the *S*(*q*) of the *F*_*T*_ (*q*) matrix. Since the vector *q* depends on *S*(*q*) of the *F*_*T*_ (*q*), it satisfies the following:

5 $$\dot{\mathbf{{q}}} = S(q){\mathbf{p}}$$

where ${\mathbf{p}} = [\theta \;\;{\mathbf{v}}\;\;\omega ]$. The nonlinear dynamic model of wheel leg robot is obtained:

6 $$(S_{T}M(q)S){\dot{\mathbf{p}}} + S_{T}(M(q)S\dot{p} + N(q,\dot{q})) \, = S_{T}J(q)\tau$$

#### Dynamics model

**Figure 8:**
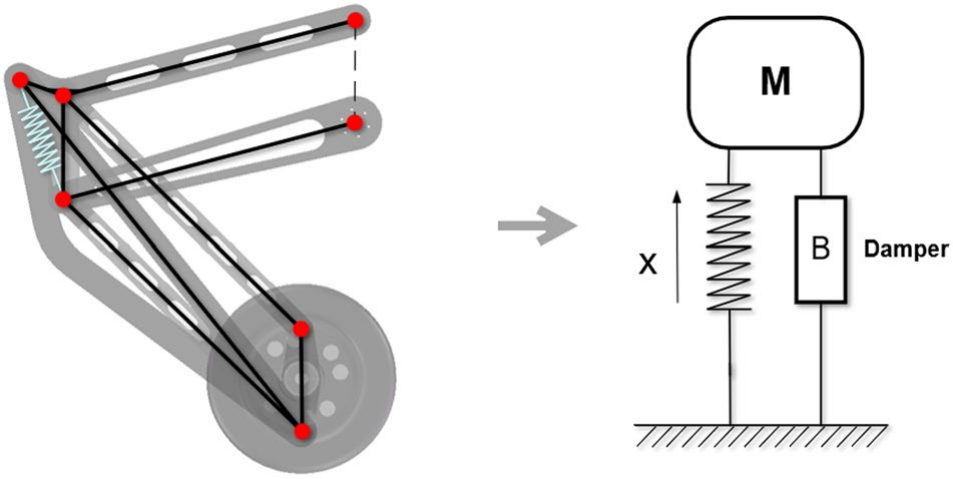
Leg system equivalent to the spring damping system.

As shown in Fig. 8, ignoring the interaction force between each link, the entire wheel-legged mechanism can be equivalent to a spring-damping system.

The dynamic equation of the equivalent spring damping system is:

7 $${\mathbf{M}}{\ddot{\text{x}}} + kx + B\dot{x} = u$$

where *k* is the elastic spring coefficient; *B* is the damping coefficient between each member; **M** is the mass matrix; *u* is the output vector of the hip joint motor.

### Control

**Figure 9:**
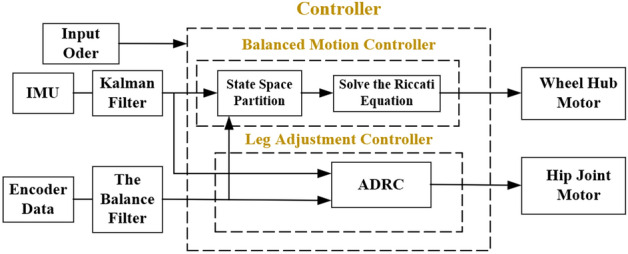
System control block diagram.

Based on the robot dynamics model, the robot control system is divided into a balance motion controller and a leg adjustment controller. The leg adjustment controller is designed by the ADRC method. On the one hand, it controls the leg structure movement to complete the standing, squatting, and other actions. On the other hand, the hip joint motor’s absolute encoder feeds back the robot’s current centroid height. The balance motion controller is realized by the LQR method, which relies on the hip joint motor to feedback on the current centroid height of the robot to complete the functions of robot balance and stable driving. Figure 9 shows the overall control block diagram of the system.

#### Body stabiling control

According to the above body dynamics model, the system’s state variable is selected as $x = [x_{{1}} \;\;x_{{2}} \;\;x_{{3}} \;\;x_{{{4}]}}^{T} = [\theta \;\;\dot{\theta }\;\;v\;\;\dot{\omega }]^{T}$, and the state space equation of the system is established. The system is linearized by Taylor expansion at the equilibrium point $x^{*} = [x_{1}^{*} \;x_{2}^{*} \;x_{3}^{*} \;x_{4}^{*} ]^{T} = 0$:

8 $$\dot{x} = {\mathbf{A}}x + {\mathbf{B}}u$$

Table 1 lists the parameters of the robot. Bring these parameters into Formula 6 to obtain the matrix **A** and **B**:

9 $${\mathbf{A}} = \left[ {\begin{array}{*{20}l} 0 \hfill & 1 \hfill & 0 \hfill & 0 \hfill \\ {f_{21} (\varphi )} \hfill & {f_{22} (\varphi )} \hfill & {f_{23} (\varphi )} \hfill & 0 \hfill \\ {f_{31} (\varphi )} \hfill & {f_{32} (\varphi )} \hfill & {f_{33} (\varphi )} \hfill & 0 \hfill \\ 0 \hfill & 0 \hfill & 0 \hfill & {f_{44} (\varphi )} \hfill \\ \end{array} } \right]$$

10 $${\mathbf{B}} = \left[ {\begin{array}{*{20}l} 0 \hfill & 0 \hfill \\ {g_{22} (\varphi )} \hfill & {g_{22} (\varphi )} \hfill \\ {g_{33} (\varphi )} \hfill & {g_{33} (\varphi )} \hfill \\ {g_{44} (\varphi )} \hfill & { - g_{44} (\varphi )} \hfill \\ \end{array} } \right]$$

**Table 1:** System parameter table.

Symbol	Meaning	Value	Units
*R*	Hub wheel motor radius	0.169	m
*L*	Distance between center of mass and axis of wheel	*Lm* (φ)	m
*mw*	Hub wheel mass	2.27	kg
*M*	Body mass	12.8	kg
*Iw*	The moment of inertia of the driving wheel about the axle	0.018	Kg m^2^
*IM*	Moment of inertia of the body	1.93	Kg m^2^
*g*	Gravity acceleration	9.8	m/s^2^
*b*	Joint dissipation energy coefficient	0.12	–
*bw*	Wheel dissipation energy coefficient	0.325	–
*IZ*	Yaw Angle moment of inertia	0.83	Kg m^2^
φ	Hip Motor angle	φ	deg

In order to simplify the model, this paper divides the state space equation into 10 segments in the height direction where φ ⊆ (70, 100), When φ = 73, 76, 79 · 100, 10 sets of state space equations corresponding to **A**1, **A**2, **A**3 ··· **A**10 and **B**1, **B**2, **B**3 ··· **B**10 are obtained, The current height of the robot fed back by the hip joint motor determines the current state space equation of the robot. Controllability analysis is performed for state spaces of different heights. The system is controllable from Formula 11:

11 $$rank(C) \, = rank([BABA_{2}BA_{3}B]) \, = {4}$$

According to the LQR control idea, the feedback gain matrix **K** is obtained by by minimizing the quadratic linear objective function **J**, Then, the linear feedback control rate **u** = −**Kx** is designed to stabilize the system at equilibrium. The quadratic performance index is:

12 $${\mathbf{J}}[{\mathbf{u}}(t)] = \frac{1}{2}\int\limits_{t0}^{\infty } {[{\mathbf{x}}^{T}(t){\mathbf{Qx}}(t) + {\mathbf{u}}^{T}(t){\mathbf{Ru}}(t)]dt}$$

*Q* ≥ 0 and *R* ≥ 0 are positive semi-definite real symmetric constant matrices in the formula and must be set according to the control weight.

According to the Lyapunov second equation, to obtain the optimal objective function, there must be a positive definite matrix **P** which is the steady-state solution of the Riccati equation. For infinite time steady state, the Riccati equation can be written as:

13 $${\mathbf{PA}} + {\mathbf{A}}_{T} {\mathbf{P}} - {\mathbf{PBR}}_{{ - 1}} {\mathbf{B}}_{T} {\mathbf{P}} + {\mathbf{Q}}(t) = 0$$

The optimal trajectory satisfies the following:

14 $$\dot{\mathbf{{x}}}(t) = ({\mathbf{A}} - {\mathbf{BR}}_{{ - 1}} {\mathbf{B}}_{T} {\mathbf{P}}){\mathbf{x}}(t)$$

From Formula 13:

15 $${\mathbf{K}} = {\mathbf{R}}_{{ - 1}} {\mathbf{B}}_{T} {\mathbf{P}}$$

The matrix **P** is obtained by iteratively solving the Riccati equation, and the feedback gain matrix **K** is obtained. Then the robot feedback control rate **u** = −**Kx** is obtained.

#### Leg motion control

**Figure 10:**
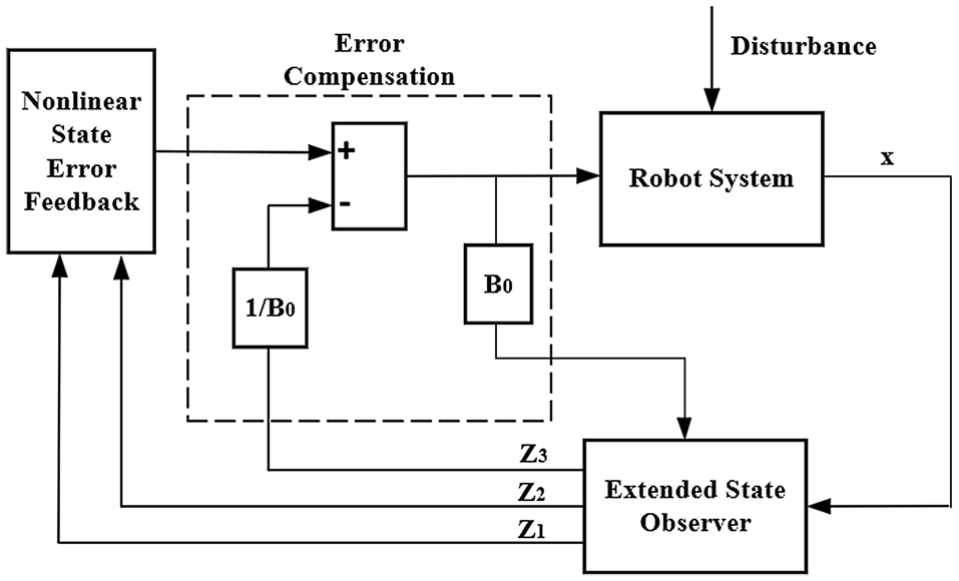
ADRC control flow diagram.

The leg adjustment controller is implemented by the ADRC method, a controller technique that estimates its compensation uncertainties. The control block diagram is shown in Fig. 10: It uses the extended state observer to compensate for the robot’s gravity disturbance and other disturbances as feedforward to the input. The robot stability is adjusted by nonlinear state error feedback.

The core idea of the ADRC controller is the extended state space observer, which observes the disturbance through the input and output of the system and eliminates the disturbance in the controller as much as possible. A second-order linear extended observer is constructed based on the spring damping system of the leg:

16 $$\left\{ \begin{aligned} & \hat{\mathbf{{z}}}(t) = {\mathbf{A}\hat{\mathbf{{z}}}}(t) + {\mathbf{Bu}}(t) + \beta ({\mathbf{y}} - \hat{\mathbf{{y}}}) \\ & \hat{\mathbf{{y}}}(t) = {\mathbf{C}\hat{{\mathbf{z}}}}(t) \\ \end{aligned} \right.$$

17 $$\left\{ \begin{aligned} & \hat{\mathbf{{z}}}_{1} = \beta _{1} ({\mathbf{y}} - \hat{\mathbf{{z}}}_{1} ) + \hat{\mathbf{{z}}}_{2} \\ & \hat{\mathbf{{z}}}_{2} = \beta _{2} ({\mathbf{y}} - \hat{\mathbf{{z}}}_{1} ) + \hat{\mathbf{{z}}}_{3} \\ & \hat{\mathbf{{z}}}_{3} = \beta _{3} ({\mathbf{y}} - \hat{\mathbf{{z}}}_{1} ) \\ \end{aligned} \right.$$

The extended state observer based on the Luenburger observer is obtained by pole assignment:

18 $$\beta_{{1}} = {3}w_{0} ,\;\beta_{{2}} = {3}w_{0}^{2} ,\;\beta_{{3}} = w_{0}^{3}$$

**Figure 11:**
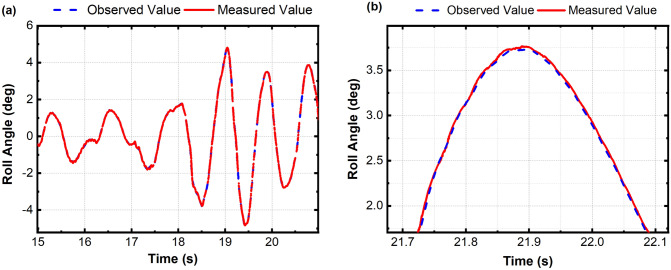
Extended state observer tracking effect diagram.

*w*_0_ is the bandwidth coefficient. By adjusting and compensating the coefficient *B*_0_, the estimated value of the observer is consistent with the actual output. The observation effect of the extended observer is shown in Fig. 11.

The pitch angle error and the velocity and angular velocity error observed by the ESO are then weighted by the nonlinear function.

19 $$u = K_{{1}} fal(e_{0} , a_{0} , \delta ) + K_{{2}} fal(e_{{1}} , a_{{1}} , \delta )$$

20 $$fal(x,a,\delta ) = \left\{ {\begin{array}{*{20}l} {\frac{x}{\delta (1 - \alpha )},\left| x \right| \le \delta } \hfill \\ {sign(x)\left| x \right|^{a} ,\left| x \right| \ge \delta } \hfill \\ \end{array} } \right.$$

where *a*, δ are the adjustment parameters; *e*_0_, *e*_1_ are the differentials of error and error respectively; *K*_1_, *K*_2_ are the gain coefficient. After nonlinear function processing, the adjustment of significant error large gain, small error small gain. It effectively solves the contradiction between rapidity and overshoot in convergence.

The regulating parameters of ADRC controller are compensation coefficient *B*_0_, bandwidth coefficient *w*_0_, *K*_1_ error gain coefficient, *K*_2_ error gain coefficient differential, and *a*, δ coefficients in the *fal* function. Usually need to fix the compensation coefficient *B*_0_ and select the smaller *K*_1_, *K*_2_, Then increase the bandwidth coefficient *w*_0_, the state observer can quickly track the error. After determining the bandwidth coefficient *w*_0_, increase *K*_1_, *K*_2_. where *a* ⊂ (1, 2), δ ⊂ (0, 1); after determining this series of parameters, increasing *B*_0_ can reduce the system’s jitter and finally select the compensation coefficient *B*_0_.

## Experiments

**Figure 12:**
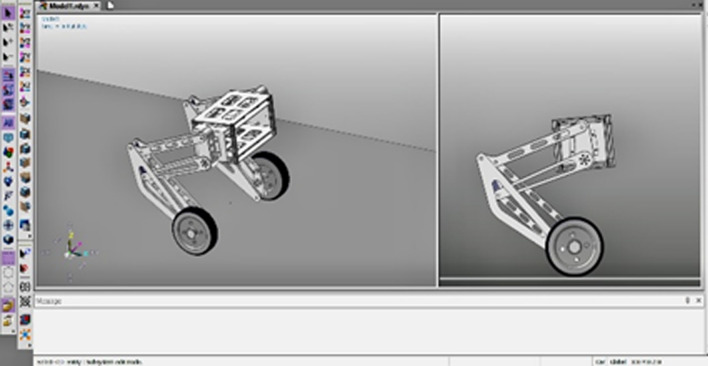
Virtual prototype of wheel-legged robot.

**Figure 13:**
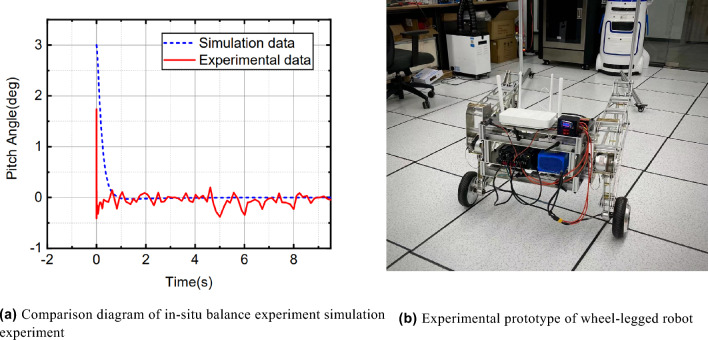
Fixed-point balance experimental diagram.

In Fig. 12 and 13b: In order to verify the performance of the wheel-legged robot control method, the research group built a simulation model and experimental prototype of the wheel-legged robot based on Recurdyn.

According to different scenarios and working conditions, the robot motion control experiments were carried out to analyze the performance of stability, speed control, and obstacle avoidance.

**Figure 14:**
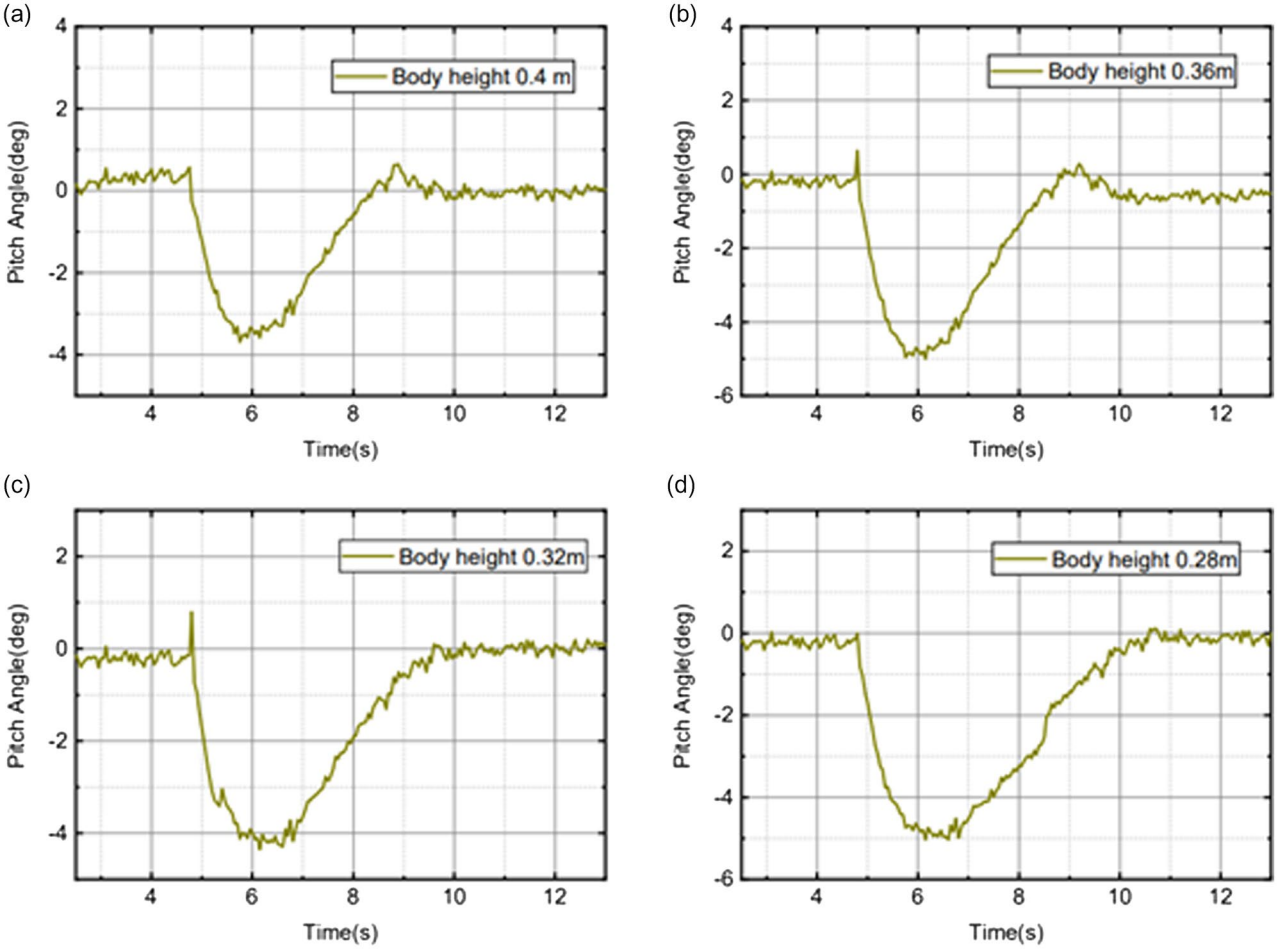
Anti-interference experimental effect diagram.

**Figure 15:**
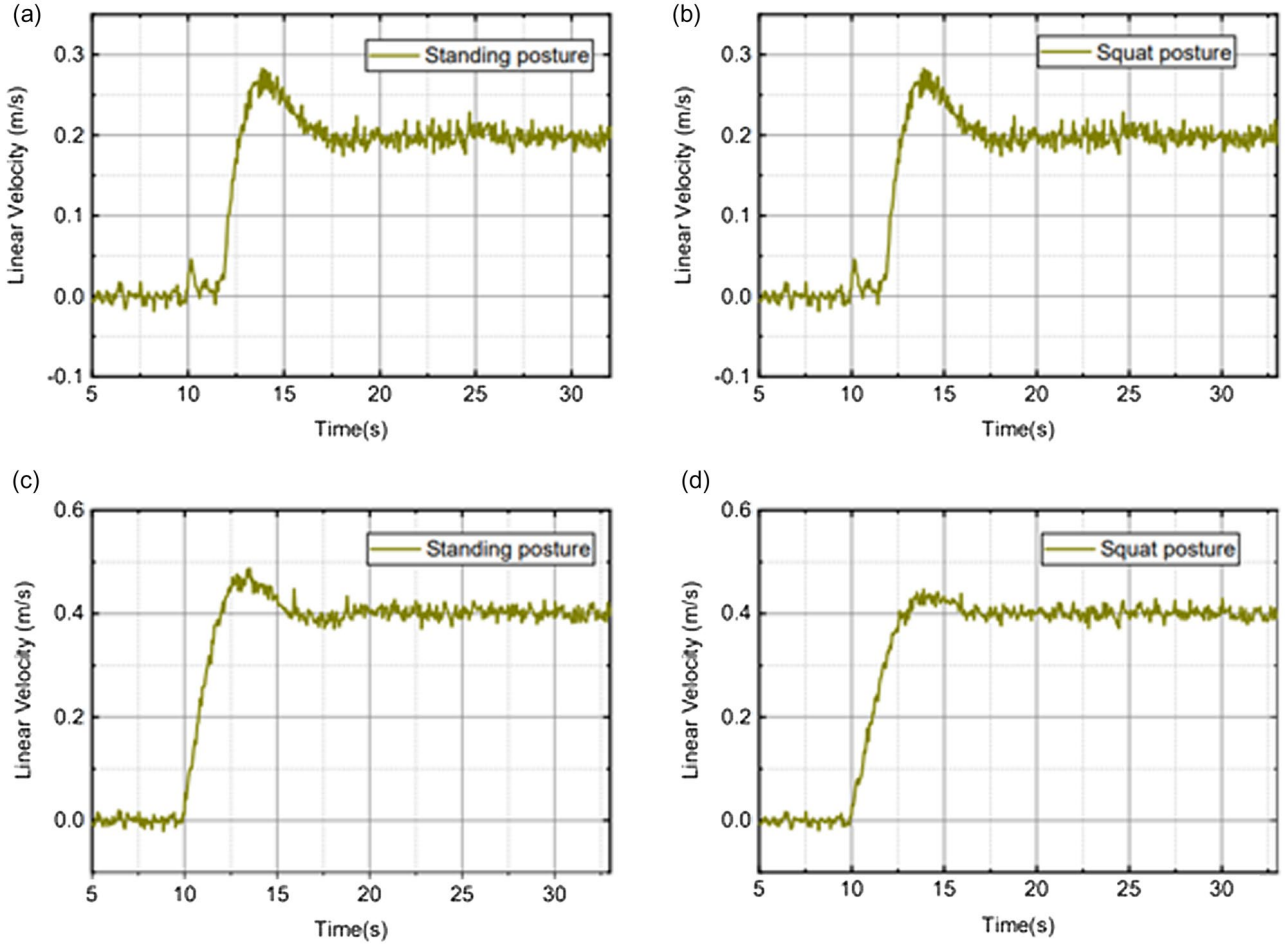
Speed tracking experiment diagram.

**Figure 16:**
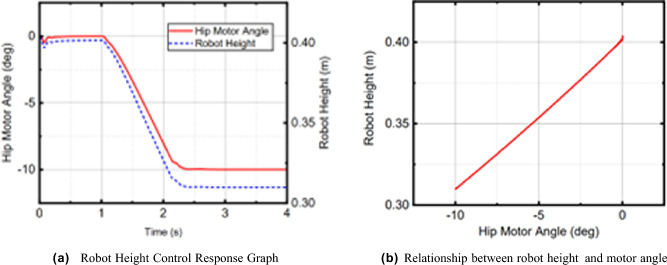
Experimental diagram of leg posture stability.

**Figure 17:**
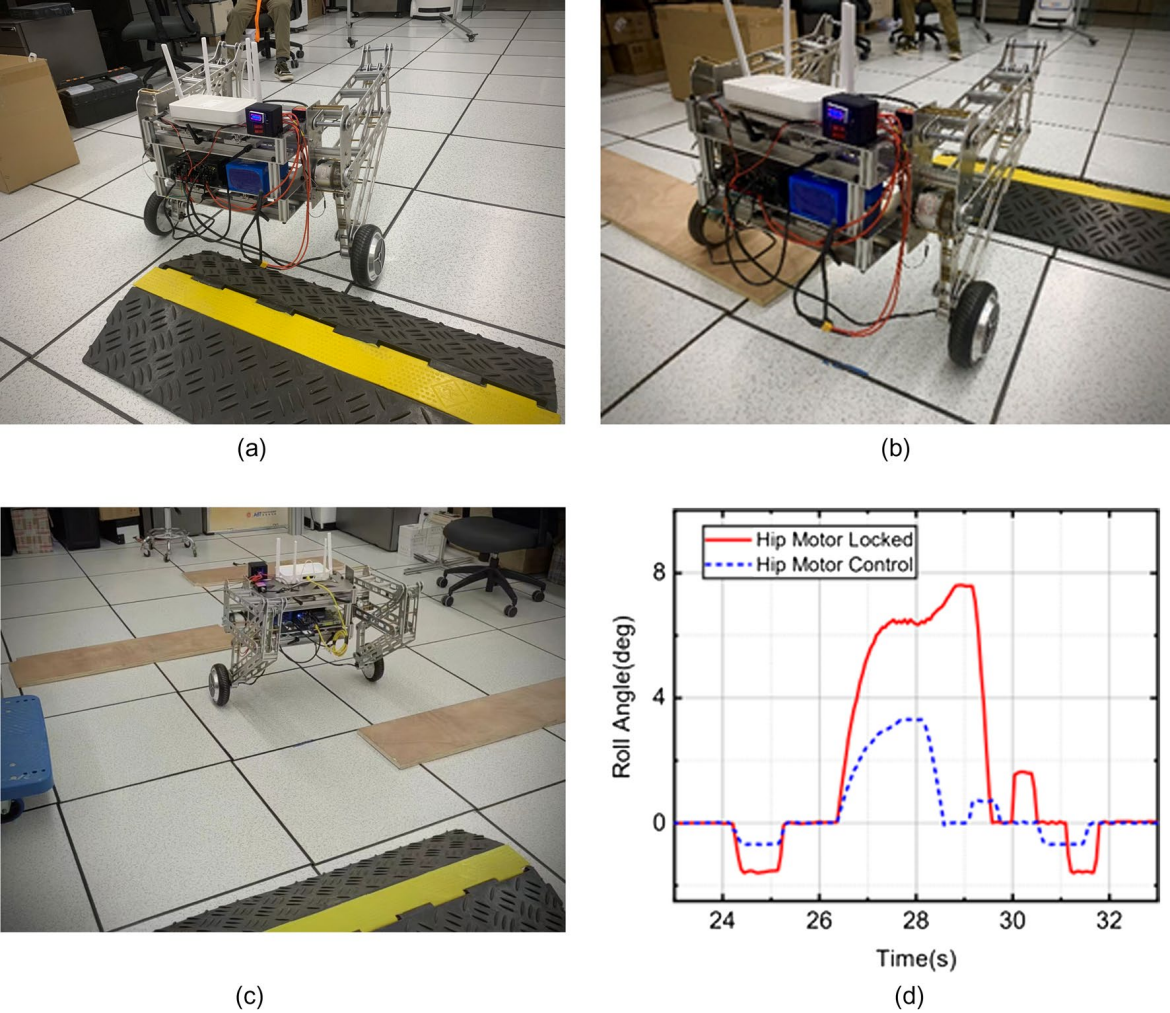
Obstacle experimental diagram.

1. Stability: Fig. 13a shows the pitch angle response of the robot in the robot simulation and experimental environments.
2. The goal of control is to maintain the robot’s balance at any time, so the free release of the stick is set to hit the robot scene and simulate the external force disturbance link. The robot flexes and stretches through the legs, and the body is at different heights. Figure 14 shows the response of the body pitch angle when the robot is subjected to impact loads at different heights. In addition to impact disturbances, other forms of disturbances, such as long-term disturbances (tension, thrust, or increased weight), can keep the robotstable.
3. Speed responsiveness: the robot needs to track the speed command sensitively at different heights. Figure 15 shows the robot’s response to the speed command when squatting (about 0.5 m) and standing (about 0.3 m). It can be seen from the diagram that different postures have accurate tracking for different speeds. In Fig. 15, the curve fluctuates greatly. This is because the IMU used in the actual measurement process has certain measurement errors, as well as the influence of mechanical structure such as the gap at the fuselage connection, resulting in certain jitter of the robot Angle. In the debugging process of the speed tracking experiment, if a larger weight coefficient is selected, the robot can track the target speed faster, but there will be a little overshoot, which is related to the friction coefficient and the inevitable error of the system. In general, such setting of parameters can make the robot perform better.
4. Posture stability: Set obstacles such as speed bumps and boards to simulate complex road conditions. The flexion and extension states of the left and right wheel legs are adjusted by detecting changes in the roll angle to accommodate changes in the terrain. First, the robot joint angle and height response to the command is shown in Fig. 16a. Figure 16b shows the change of robot height with a hip joint angle when the robot height rises at a constant speed and verifies the linear relationship between hip joint angle and robot height.
5. Figure 17 show the performance of the robot through continuous speed bump terrain with wheel or leg control. Figure 17d shows the robot’s performance through a continuous speed bump terrain with or without wheel-leg control. When the leg height is actively controlled, the robot roll angle swing is reduced by about 51.7%.

## Discussion

It is concluded that the new wheel-legged robot faces two challenges: (1) After increasing the degree of freedom of the leg, how to ensure the balancer motion control for the metamorphic center robot; (2) how to ensure stable driving when the robot encounters uneven roads.

For the first challenge, a 1-DOF seven-link leg structure is designed. Optimizing the rod length limits the lateral centroid deviation of the robot, and the robot driving and leg motion are decoupled. For the offset of the longitudinal centroid, the robot height is divided, and the feedback gain is calculated in real-time by LQR to control the balance and movement of the wheel-legged robot. For the second challenge, the ADRC controller controls the height difference between the left and right legs of the robot in real-time according to the body condition, which significantly reduces the roll angleswing amplitude of the robot and allows the robot to pass through the uneven road surface smoothly. The effectiveness and robustness of the control method are verified in various experimental results. The research results of this paper have guiding solid significance and reference value for the design of a two-wheeled word-balancing robot.

The stability control of the wheel legged robot verifies the feasibility of the robot structure design, and the seven-link structure of the leg provides a reference value for the design of the compound wheel legged robot. The stable control of the robot’s high motion provides a solid foundation for subsequent devices such as robotic arms and radar cameras. We are installing robotic arms and radars for the robot and have completed more complex tasks and environmental awareness. More details will be presented in future papers.

## Data Availability

The current study of simulation models based on Recurdyn and the original data during the experiments are available at GitHub. The Robot driver code is available from the corresponding author on reasonable request.
